# Developing a pipeline for identification, characterization and molecular editing of *cis*-regulatory elements: a case study in potato

**DOI:** 10.1007/s42994-024-00185-1

**Published:** 2024-10-30

**Authors:** Min Wan, Handan Xie, Hongwei Guo, Shenglin Jing, Deying Zeng, Bing Li, Bo Zhu, Zixian Zeng

**Affiliations:** 1 Department of Biological Science, College of Life Sciences, Sichuan Normal University, Chengdu, 610101 China; 2 Plant Functional Genomics and Bioinformatics Research Center, Sichuan Normal University, Chengdu, 610101 China; 3 Crop Research Institute, Environment-Friendly Crop Germplasm Innovation and Genetic Improvement Key Laboratory of Sichuan Province, Sichuan Academy of Agricultural Sciences, Chengdu, 610066 China

**Keywords:** *Cis-*regulatory element, Potato, CRISPR, Genome-editing

## Abstract

Crop breeding requires a balance of tradeoffs among key agronomic traits caused by gene pleiotropy. The molecular manipulation of genes can effectively improve target traits, but this may not reduce gene pleiotropy, potentially leading to undesirable traits or even lethal conditions. However, molecular editing of *cis*-regulatory elements (CREs) of target genes may facilitate the dissection of gene pleiotropy to fine-tune gene expression. In this study, we developed a pipeline, in potato, which employs open chromatin to predict candidate CREs, along with both transient and genetic assays to validate the function of CREs and CRISPR/Cas9 to edit candidate CREs. We used *StCDF1* as an example, a key gene for potato tuberization and identified a 288 bp-core promoter region, which showed photoperiodic inducibility. A homozygous CRISPR/Cas9-editing line was established, with two deletions in the core promoter, which displayed a reduced expression level, resulting in late tuberization under both long-day and short-day conditions. This pipeline provides an alternative pathway to improve a specific trait with limited downside on other phenotypes.

Dear Editor,

Crop improvement is often plagued by the tradeoff of yield and quality, mostly due to gene pleiotropy (Husaini 2022). Mutations on key genes may lead to adverse/lethal phenotypes, or cause antagonism between different phenotypes (Jiao et al. 2010; Miura et al. 2010; Tran et al. 2020). In recent years, the molecular editing of cis-regulatory elements (CRE) has provided an alternative approach to fine-tune the expression of select target genes, enabling to overcome gene pleiotropy. The editing of promoters in tomato (Rodríguez-Leal et al. 2017), maize (Liu et al. 2021) and rice (Zhou et al. 2023) not only generated continuous phenotypes, but also made possible aggregating antagonistic phenotypes. In potato, CRISPR-editing an intronic cold-responsive enhancer of a *Vacuolar Invertase* gene generated lines with phenotypic balance between normal plant growth and resistance to cold-induced sweetening (Zhu et al. 2024). Therefore, the molecular manipulation of CREs provides wide possibilities to modify the spatiotemporal pattern, the levels and the environmental responses of gene expression, facilitating the improvement of specific traits in during molecular crop breeding programs.

Potato tuberization is orchestrated by multiple genes, including the key gene *CYCLING DOF FACTOR 1* (*StCDF1*) (Kloosterman et al. 2013). In long days, StCDF1.1 can be ubiquitinated and degraded to inhibit tuberization, through its interaction with GIGANTEA (StGI) and FLAVIN BINDING KELCH REPEAT F-BOX 1 (StFKF1) (Kloosterman et al. 2013). In contrast, StCDF1.1 accumulates in short days to initiate tuberization by repressing the expression of *CONSTANS* (*StCO1/2*) and subsequently promoting expression of *SELF-PRUNING 6A* (*StSP6A*) (Abelenda et al. 2016; Kloosterman et al. 2013; Navarro et al. 2011). The natural variants of StCDF1 (StCDF1.2–1.4) also accumulate in long days to promote tuberization, due to the loss of the StFKF1 interaction site, thus leading to the adaptation of tuberization in high-latitude regions (Kloosterman et al. 2013). The expression of *StCDF1* fluctuates between day and night. It reaches the highest expression level before dawn, while it drops to the lowest expression level 8 h after dawn (Morris et al. 2013). Therefore, *StCDF1* expression at night plays a pivotal role in tuberization (Morris et al. 2013). The perception of circadian rhythm, at the transcription level, depends on the interaction between CRE of *StCDF1* and transcription factors. The ~ 3.2 kb upstream sequence of *StCDF1* was previously validated as a promoter, which responds to both photoperiod and circadian rhythms (Kondhare et al. 2019). However, the core functional promoter region, as well as the sequence associated with the response to circadian rhythm, remains unclear. Therefore, we developed a pipeline to identify, characterize and edit this CRE, and further manipulate the expression pattern of *StCDF1,* as well as the resulting tuberization time.

The pipeline consists of four main steps, including: 1) genome-wide prediction of CREs, using an open chromatin strategy detected by DNase-seq (Zeng et al. 2019), and MNase-seq (Ouyang et al. 2023) or ATAC-seq (Lu et al. 2017); 2) quick functional validation of candidate CREs using transient transformation with the firefly luciferase (LUC) reporter system in tobacco; 3) activity and tissue-specificity evaluation of CREs with the β-glucuronidase (GUS) reporter system in the native plant; 4) genome-editing of CREs, expression evaluation of target genes and phenotyping (Fig. 1A).

**Fig. 1:**
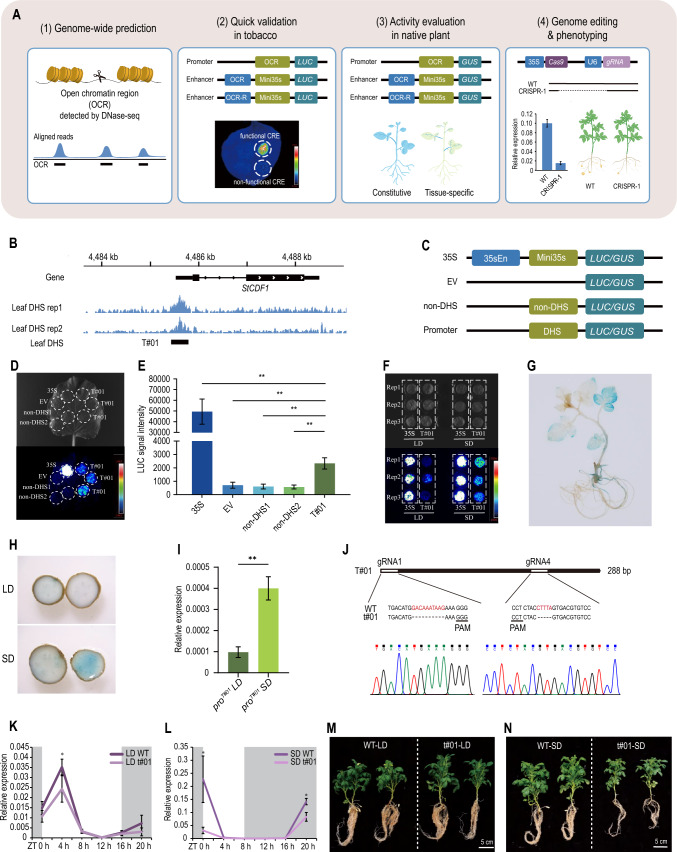
Identification, characterization and genome-editing of *cis*-regulatory elements. **A** The pipeline includes genome-wide prediction of CREs using open chromatin, quick validation of candidate CREs using transient assays, activity evaluation of CREs in the native plant using genetic transformation and editing of CREs for phenotyping. The promoter validation vector contains OCR and *LUC* or *GUS* gene. The enhancer validation vector contains OCR or reverse sequence of OCR, cauliflower mosaic virus (CaMV) minimal promoter (mini*35S*) and *LUC* or *GUS* gene. **B** Position of the candidate CRE T#01 identified from DHS using potato leaf DNase-seq data. Rep1 and rep2 were two biological replicates. **C** Diagram of *GUS* and *LUC* vector construction. The vector *35S*, containing a complete CaMV *35S* promoter and *LUC* or *GUS* gene, serves as a positive control. The vector EV only carrying the *LUC* or *GUS* gene serves as the negative control, while non-DHSs, which are randomly selected genomic DNA fragments and not in DHS, were cloned into the vector containing *LUC* or *GUS* gene as negative control, respectively. The promoter validation vector contains DHS and the *LUC* or *GUS* gene. **D** Validation of candidate CRE T#01 in tobacco. Top: bright field image, bottom: pseudo-color image. The pseudo-color image of tobacco transient transformation indicated that T#01 was predicted to be a functional promoter, based on LUC fluorescence signal intensity. **E** Semi-quantitative analysis of fluorescence intensity extracted from each sample in (**D**). Data were collected 48 h after agroinfiltration and presented as mean ± standard deviation (n = 3). **P < 0.01 in Student* t* test. **F** Promoter activity of T#01 in different photoperiods. LD represented long day with 16 h daylight/8 h darkness and SD represented short day with 8 h daylight/16 h darkness. Top: bright field image, bottom: pseudo-color image. **G** GUS signal distribution of the transgenic line carrying *proT#01::GUS* in the diploid potato DM 1–3. **H** Promoter activity of T#01 in tubers of the transgenic line detected using GUS-staining. The transgenic plants were treated under LD and SD conditions for 2 weeks, respectively. **I** *GUS* expression level of the transgenic line from (**H**). The *StEF1α* gene was used as the internal reference gene. Data are presented as mean ± standard deviation (n = 3). **P < 0.01 in Student* t* test. **J** Sequence of t#01 in the homozygous CRISPR/Cas9-edited line. PAM represents protospacer adjacent motif. **K, L** RT-qPCR analysis of *StCDF1* relative expression in leaves from WT and t#01 under LD and SD conditions. Leaf samples were taken every 4 h. “0 h” indicates zero hour after light-on. The *StEF1α* gene was used as the internal reference gene. Data are presented as mean ± standard deviation (n = 3). *P < 0.05 in Student* t* test. **M, N** Phenotype of WT (DM 1–3) and t#01 (T#01 CRISPR/Cas9-edited line) under LD and SD conditions. All plants were grown under long-day conditions for 40 days, then divided into different photoperiod for 15 days to observe tuberization

To identify the *StCDF1 *core promoter region, we utilized previously constructed DNase-seq libraries from DM 1–3 leaf tissue (Zeng et al. 2019) and detected a DNase I hypersensitive site (DHS) located across upstream and 5’UTR regions of the gene (Fig. 1B). The sequence of the DHS is 288 bp in length, designated as T#01. We constructed two sets of vectors for T#01 validation, one with the *LUC* reporter for the transient assay in tobacco and the other with the *GUS* reporter for genetic transformation in potato (Fig. 1C). The candidate CRE T#01 was directly attached upstream of the *LUC* gene for promoter function validation (Fig. 1C). In tobacco transient assays, three replicates of T#01 vector displayed apparent fluorescent signal, whereas two negative control non-DHS1 and non-DHS2 (containing non-DHS sequences) showed no signal (Fig. 1D). The semi-quantitative analysis of each signal revealed significantly higher activity of T#01 than both negative controls and empty vector (Fig. 1E), indicating that T#01 is associated with promoter activity. In addition, the vector T#01 showed stronger LUC signal under short-day (SD) condition compared to that under-long day (LD) conditions (Fig. 1F), suggesting the promoter T#01 is more active under SD than LD conditions, which is consistent with the expression pattern of *StCDF1* (Kondhare et al. 2019). Genetic transformation of the vector *proT#01::GUS* in the diploid potato DM 1–3 (with *StCDF1.1* allele) revealed that the GUS signal was mainly distributed in leaf, stem and roots of the seedlings (Fig. 1G), confirming the promoter function of T#01 and its primary activity in the leaf. Additionally, the transgenic line displayed stronger GUS signal in tubers upon a 2-week SD treatment compared to that under LD conditions (Fig. 1H), whereas the *GUS* expression level was significantly higher in tubers under SD than LD conditions (Fig. 1I), supporting the sensitivity of *StCDF1* expression to photoperiod (Kondhare et al. 2019).

To further evaluate the regulatory role of T#01 in vivo, we generated a homozygous CRISPR/Cas9-edited line with two deletions (10 bp and 5 bp) (Fig. 1J). The expression patterns of *StCDF1* in wild type (WT) and homozygous CRISPR/Cas9-edited line (t#01) showed similar fluctuation between day and night, under both LD and SD conditions (Fig. 1K, L). In both WT and t#01, *StCDF1* was expressed at a higher level before dawn and was maintained for thereafter for about 4 h after, and then declined with time (Fig. 1K, L), which is consistent with a previous study (Morris et al. 2013). Intriguingly, the CRISPR/Cas9-edited line t#01 displayed significantly lower level of *StCDF1* expression at those same time points, under both LD and SD conditions (Fig. 1K, L), suggesting that the deletions in T#01 affects the sensitivity of the sequence elements in response to light. In addition, the deletions in T#01 delayed tuberization time (Fig. 1M, N) with approximately 6 days under LD conditions and ~ 5 days under SD conditions. The deletions in T#01 also delayed flowering under LD conditions and reduced the biomass under SD conditions (Fig. 1M, N).

We have performed motif/Transcription factor (TF) searches using the 288 bp-core region (T#01) with two online database, including PlantCARE and PlantTFDB. T#01 contains a number of conserved binding sites for TF families, such as bZIP, C2H2, Dof, MYB and NAC, as well as the promoter-related sequences, such as TATA box and CAAT box. The deleted sequence in the CRISPR/Cas9-edited line t#01 was associated with multiple binding sites for TF bZIP, which are involved in positive regulation of circadian rhythm, DNA-binding TF activity, positive regulation of seed maturation, seed germination, and the abscisic acid-activated signaling pathway. These results support the promoter function of the 288 bp-core region and its potential role in photoperiod control.

This pipeline was primarily applied to characterize and edit candidate CREs, although it may not be fully optimized. For example, the evaluation of CREs in response to biotic and some abiotic stresses, such as cold, heat and drought, may not be feasible or stable in transient system in tobacco. Thus, it requires genetic transformation in potato, which take a longer time. The other main issue is how to efficiently generate homozygous CRISPR/Cas9-edited lines in potato. Thus, a genetically transformable and self-compatible germplasm is critical for this task.

## Materials and methods

### Plant materials

*Nicotiana benthamiana* and doubled monoploid potato DM 1–3 516 R44 (*Solanum tuberosum* Group phureja, 2n = 2x = 24) were used in this study. *N. benthamiana* was grown in greenhouse under the photoperiod of 16 h/24 °C daylight and 8 h/18 °C darkness. *N. benthamiana* seeds were sown on the soil and seedlings were transplants into individual pots after seedlings grew four leaves. The in vitro plantlets of potato DM 1–3 were grown on MS (Murashige and Skoog) medium with 30 g sucrose under the photoperiod of 16 h/22 °C daylight and 8 h/22 °C darkness. Wild type DM 1–3 and the CRISPR/Cas9-edited line t#01 were grown in the soil for 40 days under long day condition and divided into two groups for long-day and short-day treatment.

### Vector construction

For tobacco transient assay, DHS T#01 and non-DHSs were amplified from DM 1–3 and cloned into pCAMBIA-CRE-LUC vector containing *LUC* reporter gene using pEASY^®^-Basic Seamless Cloning and Assembly Kit (TransGen Biotech, Cat.# CU201). For DHS validation in potato, T#01 and non-DHSs were amplified from DM 1–3 and cloned into vector pKGWFS 7.0 containing *GUS* reporter gene using the same kit as the transient assay. The constructs were transferred into *Agrobacterium tumefaciens* strain GV3101 for either transient or genetic transformation. For CRISPR/Cas9 editing, four gRNAs were designed using the CRISPR-GE website (http://skl.scau.edu.cn/) (Xie et al. 2017). The synthesized gRNA was cloned into the cassette pKSE401 containing Cas9 and gRNA scaffold (Xing et al. 2014). Three CRISPR/Cas9 vectors were constructed, one containing two gRNA (gRNA1 5’-TGACATGGACAAATAAGAAA-3’, gRNA4 5’-TGGGGTGGACACGTCACTAA-3’), and the others containing single gRNA (gRNA2 5’-GGTTTGAGGGAGGGGAGAGT-3’, gRNA3 5’-GGACACGTCACTAAAGGTAG-3’, respectively).

### Tobacco transient assay

Agrobacterium-mediated transient assay in tobacco was performed following previously published procedure (Lin et al. 2019). The extended leaves from approximately 30-day-old tobacco were used for transient assay. The Agrobacterium-carrying plasmid was inoculated in LB liquid medium and cultured at 28 °C, 200 rpm until the OD_600_ reached 0.5, followed by centrifugation at 5000 × *g* for 10 min. The pellet was resuspended in agroinfltration buffer (10 mM MgCl_2_, 200 µM acetosyringone) and incubated at room temperature for two hours. The agroinfltration buffer containing Agrobacterium was injected into tobacco leaves using a 1 mL disposable sterile syringe. Samples containing target DHSs, as well as the negative and positive controls were injected in the same tobacco leaf. Each sample was evaluated in at least three leaves from different plants. After culturing at least 48 h, the fluorescein sodium solution (15 mg/mL) was applied to each agroinfltration site, which was subject to the fluorescence signal intensity detection using a chemiluminescence imaging system (Tanon-5200) with same exposure parameters.

### Potato transformation

Agrobacterium-mediated transformation in potato was performed, as previously described (Ducreux et al. 2005). Both internodes and leaves from 4 to 6 weeks in vitro plantlets were used for genetic transformation. Transgenic lines were first screened using an MS medium with 50 mg/L kanamycin. For DHS validation, the transgenic lines from the first screening were confirmed using PCR with primers 5’-GCTCATTAAACTCCAGAAACCCGCG-3’ and 5’-CGTCCTTGAAGAAGATGGTGCGC-3’. For CRISPR/Cas9 editing, transgenic lines from the first screening were treated for 3–4 cycles of 30 h at 37 °C and 42 h at 24 °C. After returning to normal conditions for 1 week, the top and lateral shoots were cut off for in vitro plantlet reproduction (LeBlanc et al. 2018). When the new plantlets grew to 10–15 cm, genomic DNA was extracted for PCR validation, using primers 5’-GACAAGAAGTACTCGATCGGCCTCG-3’ and 5’-CTCGAAGAGCTGGTTGTACGTCTGC-3’.

### GUS histochemical assay

GUS-staining steps followed published procedurse (Zhu et al. 2015) with only minor modifications, including vacuum infiltration for 1 h and incubation at 37 °C for 24 h. After staining, the GUS-stained samples were decolorized using 80% ethanol for 1 to 2 days. Images were captured using a digital camera with macro lens.

### Reverse transcription and RT-qPCR

Total RNA was extracted from potato leaves and tubers by using a OminiPlant RNA Kit (CWBIO, Cat.# CW2598). Reverse transcription was performed using 2 μg of total RNA with a EasyScript® reverse transcription kit (TransGen Biotech, Cat.# AE341). RT-qPCR was conducted a SuperStar Universal SYBR Master Mix (CWBIO, Cat.#CW3888). *StEF1α* gene was used as the internal reference gene (Zeng et al. 2019). The relative expression was calculated using 2^−∆∆Ct^ method and the significance of difference between samples were tested using the paired *t*-test.

### DNase-seq and data analysis

DNase-seq reads were obtained from our previously published data (Zeng et al. 2019). The raw reads from DNase-seq were cleaned using Cutadapt V3.7 program (Martin 2011) with a minimum base quality of 20 and a minimum base length of 4. Cleaned DNase-seq reads were aligned to the DM 1–3 genome assembly (PGSC v6.1) using Bowtie 1 with no mismatches allowed. Only reads that mapped to unique positions were used for further analysis. DNase I hypersensitive sites (DHSs) were identified using Popera with default parameters (https://github.com/forrestzhang/Popera). Motifs underlying T#01 and its potential bound transcription factors were predicted using online tools, including PlantCARE (https://bioinformatics.psb.ugent.be/webtools/plantcare/html/) and PlantTFDB (https://planttfdb.gao-lab.org/prediction.php). The 288 bp-core sequence of T#01 was used for motif and TF searching with default parameter.

## Data Availability

The datasets generated and/or analyzed during the current study are available from the corresponding author upon request.
